# A Novel *LAMA5* Variant in Adult‐Onset Focal Segmental Glomerulosclerosis: A Case Report

**DOI:** 10.1155/crig/7603821

**Published:** 2026-10-18

**Authors:** Randah Abdullah Dahlan, Roaa Hani Fairoozy

**Affiliations:** ^1^ Department of Internal Medicine King Abdullah Medical City Mecca Makkah Province Saudi Arabia kamc.med.sa; ^2^ Department of Laboratory King Abdullah Medical City Mecca Makkah Province Saudi Arabia kamc.med.sa

**Keywords:** focal segmental glomerulosclerosis, FSGS, genetic kidney diseases, *LAMA5*, nephrotic syndrome

## Abstract

Focal segmental glomerulosclerosis (FSGS) is a common histopathological diagnosis among adult patients presenting with nephrotic syndrome. This pattern is associated with diverse underlying pathophysiological mechanisms, which may influence disease management, therapeutic response, and prognosis. Genetic forms of FSGS are caused by variants in genes encoding proteins expressed in podocytes or the glomerular basement membrane. Multiple genes have been implicated in the pathogenesis of genetic FSGS, and clinical presentation can vary depending on the specific gene involved. We report the case of a young man with nephrotic syndrome and a histopathological diagnosis of FSGS, in whom genetic testing identified a novel *LAMA5* c.6940C > T p.(Arg2314Trp) variant in a consanguineous Saudi family, consistent with autosomal recessive inheritance. Family genetic analysis revealed that the proband’s mother and two siblings are homozygous for the identified variant, whereas his father and the remaining two siblings are heterozygous. The variant is currently classified as a variant of uncertain significance (VUS). This report characterizes the associated clinical phenotype and discusses the findings of family segregation analysis in the context of variable expressivity and incomplete penetrance.

## 1. Introduction

FSGS is a histopathological pattern of glomerular injury identified on light microscopy of kidney biopsy specimens. It is characterized by sclerosis affecting segments (segmental) of some (focal) glomeruli [1]. Based on clinical presentation and histopathological findings, FSGS is classified into primary, secondary, genetic, and undetermined forms [2]. This clinicopathologic classification is essential for guiding appropriate management strategies. Although these forms share the common feature of FSGS lesions on light microscopy, their pathogenesis, clinical manifestations, management, and prognosis may vary significantly [1, 2]. Primary FSGS is thought to result from an unidentified circulating factor that induces podocyte injury. It is more commonly observed in adolescents and young adults, who typically present with nephrotic‐range proteinuria, hypoalbuminemia, and hyperlipidemia [1, 2]. Electron microscopy in primary FSGS usually demonstrates diffuse foot process effacement. Patients often require immunosuppressive therapy and are at high risk of progression to end‐stage renal disease (ESRD) if untreated. Additionally, this form has a higher likelihood of recurrence following kidney transplantation [1, 2].

Secondary FSGS typically arises as an adaptive response to glomerular hypertrophy or hyperfiltration, other glomerular diseases, or direct podocyte injury (e.g., drugs or viral infections) [1, 2]. Patients generally present with subnephrotic‐range proteinuria, preserved serum albumin levels, and absence of peripheral edema [1, 2]. Electron microscopy usually shows segmental foot process effacement (less than 80%). Management primarily involves supportive therapy and addressing the underlying cause, when identifiable. Recurrence after kidney transplantation is not expected in secondary FSGS [1, 2].

Genetic FSGS is caused by variants in genes encoding proteins essential for podocyte structure or function [2]. It is more prevalent in populations with high rates of consanguinity [3–5]. Clinical presentation is variable and may include extra‐renal manifestations depending on the gene involved [2]. Genetic FSGS can present in childhood or adulthood and is typically resistant to immunosuppressive therapy. The extent of foot process effacement on electron microscopy may resemble that seen in primary or secondary FSGS. Unlike primary FSGS, genetic forms do not recur after kidney transplantation [2].

In this report, we describe an individual harboring a previously unreported *LAMA5* variant, characterize the associated clinical phenotype, and discuss the findings of family segregation analysis.

## 2. Case Report

A 20‐year‐old man was referred in November 2022 to our tertiary care center in Makkah, Saudi Arabia, for kidney biopsy. He had developed facial and lower limb swelling 5 months prior to referral. He initially presented to a nearby hospital, where he was diagnosed with nephrotic syndrome and started on oral prednisone and cyclosporine, and was subsequently referred to our center for further evaluation and kidney biopsy. At presentation to our clinic, his only complaints were persistent facial puffiness and lower limb edema. He had no history of chronic medical illnesses, prior surgeries, or known drug allergies. His parents are first‐degree cousins and are both reportedly healthy, with no chronic medical conditions. He has three brothers and one sister, all of whom are healthy, and there is no known family history of renal disease. In addition to prednisone 10 mg daily and cyclosporine 50 mg twice daily, his medications included ramipril 5 mg daily, calcium carbonate 500 mg daily, atorvastatin 20 mg daily, and weekly vitamin D supplementation. He was a university student, a nonsmoker, and single. On physical examination, his blood pressure was 139/88 mmHg. He had facial puffiness and bilateral lower limb edema. The remainder of the examination was unremarkable. His initial laboratory investigations are summarized in Table 1.

**TABLE 1:** Index case initial investigations.

Test item	Reference range	Result
Hemoglobin (g/dL)	13.5–17.5	16.8
Platelet (x 10^9^/L)	150–400	408
White blood cells (x 10^9^/L)	3.9–11	14.7
Sodium (mmol/L)	136–145	140
Potassium (mmol/L)	3.5–5.1	4.2
Bicarbonate (mmol/L)	22–29	24.0
Blood urea nitrogen (mg/dL)	8–24	16
Creatinine (mg/dL)	0.74–1.35	0.59
Corrected Calcium (mg/dL)	8.5–10.1	9.4
Phosphorus (mg/dL)	2.5–4.5	5.5
Parathyroid Hormone (pg/mL)	15–68.3	27.8
Hemoglobin A1C (%)	< 5.8	5.6
Albumin (g/dL)	3.5–5.2	2.9
Hepatitis B virus surface antigen	Nonreactive	Nonreactive
Hepatitis C virus antibodies screen	Nonreactive	Nonreactive
Human immunodeficiency virus screening antigen‐antibody test	Nonreactive	Nonreactive
Antinuclear antibodies–ELISA (units)	< 20	4.03
Anti‐dsDNA antibodies (IU/mL)	0–200	19.8
Anti‐glomerular basement membrane antibodies (U/mL)	< 7	< 2
C3 (mg/dL)	79.0–152.0	194
C4 (mg/dL)	16.0–38	42.8
Urine Protein Creatinine Ratio (mg/mg)	< 0.18	7.51
24‐hours urine protein (mg/24 h)	< 150.00	5015.25
Urine examination: bland with no RBCs or WBCs or casts, +3 protein		
Ultrasound kidneys: Both kidneys show normal size, surface, shape with mild increased echogenicity. There is good corticomedullary differentiation		

A kidney biopsy was subsequently performed. Light microscopic examination of 28 glomeruli revealed mild mesangial matrix expansion and hypercellularity, with normal capillary wall thickness and patent capillary lumina. Eight glomeruli demonstrated segmental sclerosis, some involving the tip domain. None of the glomeruli showed global sclerosis. There was minimal interstitial fibrosis and tubular atrophy, accompanied by a mild interstitial mononuclear inflammatory infiltrate admixed with a few eosinophils and foam cells. The arteries and arterioles were unremarkable. Immunofluorescence studies showed no significant glomerular staining for IgG, IgA, IgM, C3, C1q, fibrinogen, or kappa and lambda light chains. No extraglomerular staining was identified. Electron microscopy demonstrated glomerular basement membranes of normal thickness, contour, and texture. The mesangial areas were unremarkable, and no definite electron‐dense deposits were identified. The podocytes exhibited diffuse foot process effacement. Overall, the histopathological findings were consistent with a diagnosis of focal segmental glomerulosclerosis (FSGS).

During subsequent clinic visits, it was noted that the proteinuria persisted and worsened despite an increased dose of steroids, ongoing cyclosporine therapy (target trough level 100–175 ng/mL), the addition of empagliflozin, and treatment with an angiotensin receptor blocker. Resistance to both steroids and cyclosporine, along with a positive history of consanguinity, raised suspicion for a genetic form of FSGS. Therefore, molecular genetic analysis for FSGS was requested.

Genetic testing was performed at BioScientia International Laboratory Center in Frankfurt, Germany, using next‐generation sequencing (NGS). The panel included the following genes: *ACTN4, ANLN, APOL1, ARHGAP24, ARHGDIA, ATF3, AVIL, BLVRB, CD2AP, COL4A3, COL4A4, COL4A5, COQ2, COQ6, COQ8B, CRB2, CUBN, DAAM2, DGKE, DLC1, EMP2, FAT1, FN1, GON7, INF2, ITGA3, ITGB4, ITSN1, KANK1, KANK2, KANK4, LAGE3, LAMB2, LMX1B, LRP2, MAGI2, MT-TL1, MYH9, MYO1E, NPHS1, NPHS2, NUP107, NUP133, NUP160, NUP205, NUP85, NUP93, OCRL, OSGEP, PAX2, PDSS2, PLCE1, PTPRO, SCARB2, SGPL1, SMARCAL1, TBC1D8B, TNS2, TP53RK, TPRKB, TRPC6, TTC21B, WDR4, WDR73, WT1, XPO5, and YRDC.* However, no pathogenic or likely pathogenic variant was identified in any of the tested genes.

Subsequently, exome sequencing was performed and identified a homozygous *LAMA5* variant [NM_005560.6:c.6940C > T; p.(Arg2314Trp)]. Although *LAMA5* is associated with autosomal recessive nephrotic syndrome, the identified variant has not been previously reported in the literature and was therefore classified as a variant of uncertain significance (VUS; ACMG/AMP class III) according to the 2015 the American College of Medical Genetics and Genomics and Association for Molecular Pathology (ACMG/AMP) standards and guidelines (Table 2) [6].

**TABLE 2:** Genetic test results of the index case.

Gene (isoform)	Phenotype MIM number (mode of Inheritance)	Variant	Zygosity	MAF gnomAD (%)	Classification
LAMA5 (NM_005560.6)	620049 (AR)	c.6940C > T p.(Arg2314Trp) chr20:60894001	homozygous	0.038	uncertain significance

*Note:* gnomAD, Genome Aggregation Database.

Abbreviations: AR, autosomal recessive; MAF, minor allele frequency; MIM, Mendelian Inheritance in Man.

To investigate the hereditary nature of the variant, family segregation analysis was performed. Family members presented to a private laboratory (Al Borg Diagnostics, Saudi Arabia) for targeted Sanger sequencing of the relevant *LAMA5* regions (reference sequence: NM_005560.6). Genetic analysis revealed that the proband’s mother and two siblings were homozygous for the identified variant, while the father and the remaining two siblings were heterozygous for the *LAMA5* c.6940C > T; p.(Arg2314Trp) variant (Figure 1). Sanger sequencing results are presented in Figure 2.

**FIGURE 1:**
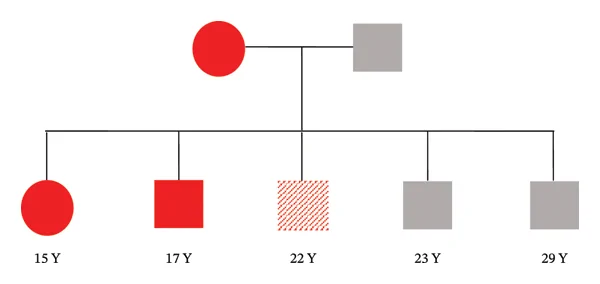
Pedigree of the patient’s family. The diagonally hatched red square represents the index case. Red shapes represent family members who were homozygous for the identified *LAMA5* variant, whereas grey shapes represent family members who were heterozygous for the same variant. Numbers listed below the shapes indicate the age of each individual in years. Circles represent women/girls and squares represent men/boys. Pedigree symbols represent gender.

**FIGURE 2:**
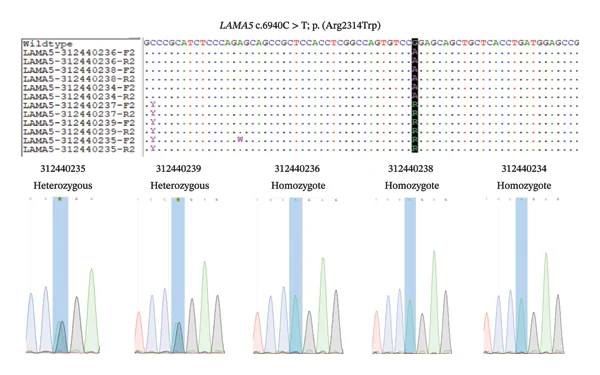
Sanger sequencing analysis of exon 52 of the *LAMA5* gene was performed in the proband and his tested family members. The electropherogram shows a base change, distinguishing homozygous from heterozygous patterns associated with *LAMA5*:c.6940C > T; p.(Arg2314Trp) mutations.

While awaiting the genetic testing results, cyclosporine was discontinued and replaced with mycophenolate mofetil at a dose of 1 g twice daily; however, proteinuria did not improve (Table 3). After 8 months, a decision was made to initiate a low‐dose multitarget regimen consisting of prednisone 5 mg daily, mycophenolate mofetil 500 mg twice daily, and cyclosporine 50 mg twice daily. Notably, proteinuria subsequently began to improve progressively (Table 3).

**TABLE 3:** Serial 24‐hour urine protein results in the index case.

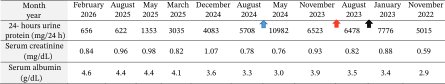

*Note:* Black arrow indicates the time when the prednisone dose was increased to 60 mg/day while cyclosporine was continued. Red arrow indicates the time when cyclosporine was switched to mycophenolate mofetil. Blue arrow indicates the time when multi‐target therapy was initiated.

Despite the genetic testing results, this multitarget therapy was continued, with a reduction in the dose of mycophenolate mofetil and cyclosporine. At the most recent follow‐up in February 2026, the individual was asymptomatic and had proteinuria of less than 1 g/day. His medications at that time included amlodipine 10 mg daily, calcium carbonate 600 mg once daily, cyclosporine 50 mg once daily, empagliflozin 25 mg daily, irbesartan 300 mg daily, mycophenolate mofetil 500 mg daily, omeprazole, prednisone 5 mg daily, and atorvastatin.

All family members were evaluated by their primary physicians and underwent laboratory testing. With the exception of the mother, all had normal creatinine, albumin, and urine protein‐to‐creatinine ratios. The mother had a mildly elevated urine protein‐to‐creatinine ratio of 28.5 mg/mmol (reference range, 1–14 mg/mmol), with normal albumin, creatinine, and glycosylated hemoglobin levels.

### 2.1. Pathogenicity of the Identified Variant

To classify the identified *LAMA5* variant, we applied the 2015 ACMG/AMP standards and guidelines [6]. The applicable classification criteria are summarized in Table 4. Based on this framework, the *LAMA5* c.6940C > T; (p.Arg2314Trp) variant remains classified as a VUS. The variant was identified at a low frequency in population databases, including gnomAD, and the available clinical, genetic, and computational evidence was insufficient to establish pathogenicity. Computational prediction tools do not consistently support a deleterious effect. The presence of the homozygous variant in clinically unaffected family members further limits the strength of the segregation evidence. Additional functional studies and identification of unrelated affected individuals harboring the same variant are needed to clarify its clinical significance.

**TABLE 4:** ACMG/AMP criteria considered for the classification of the *LAMA5* c.6940C > T (p.Arg2314Trp) variant.

ACMG criterion	Evidence and justification
PM2 (Pathogenic, Moderate)—considered, not applied	The variant is present at a low frequency in population databases, including gnomAD, and therefore does not meet the original PM2 criterion of being absent from population databases. In gnomAD, the variant has an overall allele frequency of 0.0003476 (555/1,596,864 alleles) and has been observed in the homozygous state in one individual. The allele frequency is higher in the Middle Eastern population (0.005474; 33/6028 alleles). Therefore, PM2 was considered but not applied, as the available population data do not support the required absence of the variant from population databases.
PP3—considered, not applied	The variant results in a nonconservative amino acid substitution, replacing arginine, a positively charged basic residue, with tryptophan, a bulky hydrophobic aromatic residue, at codon 2314. This substitution could potentially affect local protein conformation, intermolecular interactions, or extracellular matrix assembly, given that *LAMA5* encodes laminin *α*5, a key structural component of the glomerular basement membrane. However, computational evidence is predominantly consistent with a benign or tolerated effect. The CADD score (18.1) suggests moderate evolutionary constraint but remains below the commonly used threshold for stronger evidence of deleteriousness, whereas REVEL, PolyPhen‐2, SIFT, and SpliceAI predict a benign or tolerated effect with no significant predicted effect on splicing. Overall, the computational evidence does not provide sufficient support for a deleterious effect; therefore, PP3 was not applied.
PP4—considered, not applied	The proband presented with steroid‐resistant nephrotic syndrome and FSGS on kidney biopsy in the setting of parental consanguinity. This phenotype is consistent with the reported clinical spectrum of autosomal recessive *LAMA5*‐associated nephropathy; however, it is not sufficiently specific to establish the diagnosis based on phenotype alone. Therefore, PP4 was considered but not applied.
PP1 (Pathogenic, Supporting)—considered, not applied	The variant is present in the homozygous state in the proband. However, the presence of the same homozygous variant in the mother and two clinically unaffected siblings provides unfavorable segregation evidence. Therefore, the available family data do not support application of PP1.
BS4 (Benign, Strong)—considered, not applied	Although the presence of the variant in homozygous unaffected family members (the mother and two siblings) would ordinarily suggest lack of segregation with the disease phenotype, the BS4 criterion was not applied because the clinical significance of the unaffected homozygous relatives cannot be determined with certainty from the available family data. Factors such as age‐dependent or reduced penetrance and variable expressivity may complicate interpretation of the segregation findings; however, these mechanisms cannot be established in the present family. Accordingly, BS4 was considered but not applied, and the unfavorable segregation finding is acknowledged as a limitation of the evidence supporting pathogenicity.

*Note:* PM2, absence or rarity in population databases; PP3, computational evidence supporting a deleterious effect; PP4, phenotype or family history highly specific for a disease; PP1, cosegregation with disease; BS4, lack of segregation with disease.

Abbreviations: ACMG/AMP, American College of Medical Genetics and Genomics/Association for Molecular Pathology; CADD, Combined Annotation Dependent Depletion; REVEL, Rare Exome Variant Ensemble Learner; SIFT, Sorting Intolerant From Tolerant; SpliceAI, Splicing Artificial Intelligence.

## 3. Discussion

The prevalence of genetic FSGS varies according to several demographic and epidemiological factors. Notably, it is strongly influenced by the age at disease onset, with monogenic causes identified in up to 50% of infantile‐onset cases, decreasing to approximately 10%–20% in adolescent and adult‐onset disease [3]. Additionally, a higher prevalence of genetic FSGS has been observed in regions where consanguinity is more common, reflecting the increased likelihood of autosomal recessive inheritance in these populations [3–5]. However, the prevalence of adult‐onset genetic FSGS is likely underestimated due to the limited number of available studies [6].

Most patients with childhood‐onset genetic FSGS harbor autosomal recessive variants, which are typically fully penetrant and present with severe nephrotic syndrome [2]. Extra‐renal manifestations may also occur, depending on the specific genetic cause involved [2]. In contrast, the clinical and histological features of adult‐onset genetic FSGS are less well characterized [7]. Adult‐onset genetic FSGS is more commonly inherited in an autosomal dominant pattern with variable penetrance, and affected individuals may present with a broad spectrum of proteinuria and kidney disease severity [7].

To date, more than 50 genes have been implicated in the pathogenesis of monogenic forms of FSGS, reflecting the marked genetic heterogeneity of this condition [1, 2, 8–10]. These genes encode proteins that are essential for the proper function of the glomerular filtration barrier and can be broadly categorized into several functional groups, including slit diaphragm–associated proteins (e.g., *NPHS1* and *NPHS2*), actin cytoskeleton and regulatory proteins (e.g., *ACTN4* and *ANLN*), nuclear pore complex proteins (e.g., *NUP93* and *NUP205*), cell membrane–associated proteins (e.g., *PTPRO* and *EMP2*), and components of the glomerular basement membrane (e.g., *LAMA5*) [10]. The specific protein affected influences the severity of podocyte injury and, consequently, the clinical and histopathological manifestations of the disease [10]. Furthermore, depending on the gene involved and its biological role, environmental and epigenetic modifiers may contribute to phenotypic variability among family members carrying the same genetic variant [1].

The *LAMA5* gene encodes the laminin *α*5 chain, a major component of laminin‐521 in the glomerular basement membrane that plays an important role in maintaining glomerular structure and podocyte adhesion [11, 12]. Disruption of laminin *α*5 may impair the structural and functional integrity of the glomerular filtration barrier, potentially contributing to podocyte injury, proteinuria, and progressive glomerular disease [11, 12]. While *LAMA5*‐related disease has been classically described in pediatric populations presenting with nephrotic syndrome, emerging evidence indicates that *LAMA5* variants can also manifest in adulthood, with a spectrum of phenotypes ranging from isolated proteinuria to FSGS and chronic kidney disease [11–13]. However, the full phenotypic spectrum of *LAMA5*‐associated kidney disease in adults’ remains incompletely characterized [12]. Reported phenotypes may vary according to the type and location of the underlying variant [12, 14]. Biallelic truncating variants have generally been associated with severe, early‐onset nephrotic syndrome, whereas missense variants have been reported in individuals with milder and later‐onset phenotypes [12, 14]. This genotype–phenotype spectrum provides biological context for the possibility of variable clinical expression associated with missense *LAMA5* variants; however, it does not establish the pathogenicity of the variant identified in the present case.

Previous reports have also highlighted the difficulty of establishing the clinical significance of homozygous *LAMA5* variants [13]. Braun et al. reported homozygous *LAMA5* variants classified as VUS in three consanguineous families with pediatric nephrotic syndrome, in whom variants in established disease‐associated genes had been excluded and a recessive monogenic cause was strongly suspected based on the pedigree [13]. Although affected individuals shared a phenotype of nephrotic syndrome, considerable variability was observed in clinical presentation, response to immunosuppressive treatment, and progression to end‐stage kidney disease. The authors considered the *LAMA5* variants possible inherited contributors to nephrotic syndrome but emphasized that additional evidence and functional studies were required to establish their clinical significance [13]. These observations provide relevant context for the present case, while emphasizing that the presence of a homozygous *LAMA5* variant alone does not establish a causal relationship.

Determining the genetic basis of FSGS is crucial for clinical management and genetic counseling. First, it may help predict the likelihood of response to immunosuppressive therapy, as many genetic forms of FSGS currently lack disease‐specific treatments [2]. Recognizing a genetic etiology may therefore support reassessment of the need for ongoing immunosuppressive therapy in individuals who fail to respond. However, it is important to note that selected genetic subtypes may still respond to immunosuppressive agents, while others may benefit from targeted therapies such as vitamin or cofactor supplementation [2, 15]. Novel and gene‐based therapies also represent promising future treatment options [2].

Establishing a genetic diagnosis is additionally important for predicting the risk of disease recurrence after kidney transplantation. In general, the recurrence rate of FSGS in patients with primary (idiopathic) disease ranges from approximately 30% to 70%, whereas it is markedly lower in patients with genetic FSGS [2].

Furthermore, identifying a genetic cause enables screening and counseling of at‐risk family members, providing an opportunity for early intervention and secondary prevention of disease progression. This may include optimization of blood pressure control and initiation of antiproteinuric therapies. In populations where consanguinity is common, appropriate genetic counseling may also be considered for family members.

In the present case, exome sequencing identified a homozygous *LAMA5* c.6940C > T; p.(Arg2314Trp) missense variant in an individual with adult‐onset nephrotic syndrome and FSGS. Although the variant is compatible with the reported autosomal recessive inheritance pattern of *LAMA5*‐associated nephropathy [12, 13], its clinical significance remains uncertain, and it is currently classified as a VUS according to the 2015 ACMG/AMP standards and guidelines. Several findings limit the evidence supporting a pathogenic role for this variant. The variant is present at a low frequency in population databases, including gnomAD, and therefore does not meet the original PM2 criterion. Furthermore, the available computational evidence does not provide sufficient support for a deleterious effect. The ACMG/AMP criteria considered in the classification of the variant, together with the evidence supporting and limiting their application, are summarized in Table 4.

The segregation findings also limit the evidence for pathogenicity, as the same homozygous variant was identified in the mother and two clinically unaffected siblings. Although incomplete penetrance and variable expressivity may provide possible explanations for this observation, these mechanisms cannot be established from the present family alone and should not be interpreted as evidence of pathogenicity [16, 17]. Phenotypic variability among individuals carrying the same genetic variant may also be influenced by genetic background, polygenic factors, and environmental modifiers [16, 17]. Such factors could theoretically contribute to differences in disease expression among members of the same family; however, no polygenic risk assessment or specific modifier analysis was performed in the present family, and these possibilities therefore remain hypothetical. The segregation findings and their implications for ACMG/AMP classification are summarized in Table 4. Taken together, the population‐frequency, computational, and segregation findings do not provide sufficient evidence to establish a pathogenic role for the identified variant. Further studies involving unrelated affected individuals, functional assessment of the variant, and additional segregation data will be required to determine whether the variant contributes to kidney disease.

The individual did not initially respond to high‐dose corticosteroids or therapeutic‐dose cyclosporine. However, a gradual and marked improvement in proteinuria was subsequently observed following the initiation of a multitarget immunosuppressive regimen, consisting of low‐dose corticosteroids, cyclosporine, and mycophenolate mofetil. The subsequent improvement in proteinuria should be interpreted cautiously and cannot be attributed to the *LAMA5* variant, particularly given its VUS classification and the lack of evidence establishing a causal relationship. The response may reflect the effect of immunosuppressive therapy, concomitant antiproteinuric treatment, or the natural fluctuation of proteinuria. The individual was also treated with an angiotensin receptor blocker and a sodium–glucose cotransporter 2 (SGLT2) inhibitor.

Although the response to the multitarget regimen is clinically noteworthy, this single observation does not establish that individuals with *LAMA5* variants respond to this treatment approach. Further clinical experience and studies would be required before any association between *LAMA5*‐related disease and response to multitarget immunosuppression could be proposed.

The family findings also have implications for genetic counseling. Given that the same homozygous variant was identified in clinically unaffected family members, the clinical significance of homozygosity for this variant cannot be predicted with certainty. Therefore, homozygous family members should not be assumed to be either definitely affected or definitely unaffected solely on the basis of their genotype. Appropriate genetic counseling and clinical follow‐up may be considered, particularly if renal abnormalities or proteinuria develop.

In summary, this report describes a previously unreported *LAMA5* c.6940C > T; p.(Arg2314Trp) variant in an individual with adult‐onset nephrotic syndrome and FSGS. The case shares several features with previously reported *LAMA5*‐associated nephropathy, including nephrotic syndrome, FSGS on kidney biopsy, adult presentation, and a homozygous variant identified in the setting of parental consanguinity [5, 7, 11]. However, the presence of the same homozygous variant in the mother and two clinically unaffected siblings, together with the population frequency and computational findings, limits the evidence for pathogenicity. The variant therefore remains classified as a VUS.

The case highlights the challenges of interpreting *LAMA5* variants in adult‐onset FSGS, particularly in consanguineous families. It also underscores the importance of integrating population‐frequency data, computational predictions, clinical phenotype, and family segregation when applying ACMG/AMP criteria. Further functional studies and identification of unrelated affected individuals carrying the same variant will be important to clarify its clinical significance.

## Funding

There was no external financial support for this study.

## Consent

A written informed consent was obtained from the index case for the publication of this case report and the accompanying images.

## Conflicts of Interest

The authors declare no conflicts of interest.

## Data Availability

The data that support the findings of this study are available from the corresponding author upon reasonable request.
